# Analysis of translucency parameter and fluorescence intensity of 5 resin composite systems

**DOI:** 10.4317/jced.61174

**Published:** 2024-01-01

**Authors:** Ronaldo Hirata, João-Luiz-Bittencourt de Abreu, Ernesto-Byron Benalcázar-Jalkh, Pablo Atria, Álvaro-Ferrando Cascales, Jon-Salazar Cantero, Camila-Sobral Sampaio

**Affiliations:** 1 Biomaterials Division, NYU College of Dentistry, New York, NY, USA; 2 Department of Dental Clinic, School of Dentistry, Federal University of Rio de Janeiro, R. Prof. Rodolpho Paulo Rocco, 325, 21941617, Rio de Janeiro, RJ, Brazil; 3 Department of Prosthodontics and Periodontology, University of São Paulo - Bauru School of Dentistry, Bauru, SP, Brazil; 4 New York University Grossman School of Medicine, New York University; 5 Cátedra de Formación e I+D en Odontología Clinica – UCAM; 6 Department of Cariology and Comprehensive Care, NYU College of Dentistry

## Abstract

The natural outcome of dental composite restorations highly depends on the translucency of the enamel layer and fluorescence. This study aimed to evaluate the Translucency Parameter (TP) and Fluorescence Intensity (FI) of five different resin composite systems. Seven discs of each composite brand were prepared in a circular increasing thickness. For TP, a spectrophotometer measured the samples’ colors. The color difference within the white/black backgrounds obtained the translucency parameter. For FI, samples were exposed to UV light, and ten photographs per group were taken. Each specimen was analyzed digitally. A mixed model analysis to a 95% confidence level analyzed groups differences. Higher values of TP were observed for ED and EL, followed by FZ. The lowest values were observed for EO and FO. FI values descending order was EL>FO>EO>ED>FZ. The composition of fillers and organic matrix influenced the behavior of fluorescence and translucency of resin composites.

** Key words:**Resin composite, fluorescence, color, translucency parameter.

## Introduction

Resin composites are the most used restorative materials for direct restorations. Mechanical properties and optical behavior benefit the material used in anterior and posterior teeth, offering a long-term evaluation success (1-3). Its range of clinical indications, adhesive properties associated with dental adhesives, and possible repair or replacements push clinical usage in daily practice.

Color matching is a critical aspect of using resin composites in dentistry. The goal is to select a resin composite material with color and shade closely resembling a patient’s natural teeth, resulting in a seamless and aesthetically pleasing restoration.

Natural results of composite restorations depend on the optical properties of different opacities and translucencies used in the layers (1,4,5). Adequate opacities of dentin/opaque composite layers used in a correct thickness can block the oral cavity background (6). Opaque composites reproduce the natural dentin’s opaque properties, while enamel shades reproduce the enamel’s translucency. Translucency is an intermediate stage between total opacity and transparency, and enamel composites vary this behavior brand broadly to brand (1) and influence the final color of the composite (7). Final restorations’ greyish and whitish effect often comes with dentin and enamel shades translucency mistakes.

The capacity of masking the background is defined as color masking used as a reference of the relative translucency parameter (8), evaluated by the ΔE of the resin composite over black and white backgrounds obtained with a spectrophotometer (9). This method was also applied in our tests.

Besides translucency aspects of restorative materials, the natural tooth structure presents visible light emission when exposed to ultraviolet rays, called fluorescence (10-12). This behavior makes teeth appear brighter, presenting a whitish-blue appearance (13), and dentin substrate presents stronger fluorescence intensities than enamel (14).

Restorative materials should reproduce the same properties; otherwise, a low luminosity aspect would be presented, for instance, during the day and at nightclubs under the black light (15,16). Ideally, composites’ fluorescence intensities should be similar to natural dentin and enamel, even after aging (17). Inorganic fillers such as luminophore agents like ytterbium, cerium, europium, and terbium were included in the composition to provide a fluorescence behavior (10,11).

Fluorescence can be measured using a fluorescence spectrometer (17,18), photography attached to a UV illumination (19), a direct spectrometry (20), and a monochromator-based multi-mode reader (21). Our test was conducted using UV light emission captured by a digital camera and data with specific analysis of values of red (R), green (G), and blue (B) displayed field (22).

This study aimed to analyze the translucency parameter (TP) and fluorescence intensities (FI) of five composite resin systems. The null hypothesis is that all composite systems will have no difference between translucency and fluorescence levels.

## Material and Methods

Materials tested are listed in Table 1. Groups were divided into: Group EO: Estelite Omega EA2 (Tokuyama, Tokyo, Japan), Group FZ: Filtek Z350XT A2E (3M Oral Care, Saint Paul, MN, US), Group ED: Empress direct A2 enamel (Ivoclar Vivadent, Schaan, Lichtenstein), Group FO: Forma A2E (Ultradent, Indaiatuba, Sao Paulo, Brazil), Group EL: Essentia LE (GC, Tokyo, Japan).

**Table 1:**
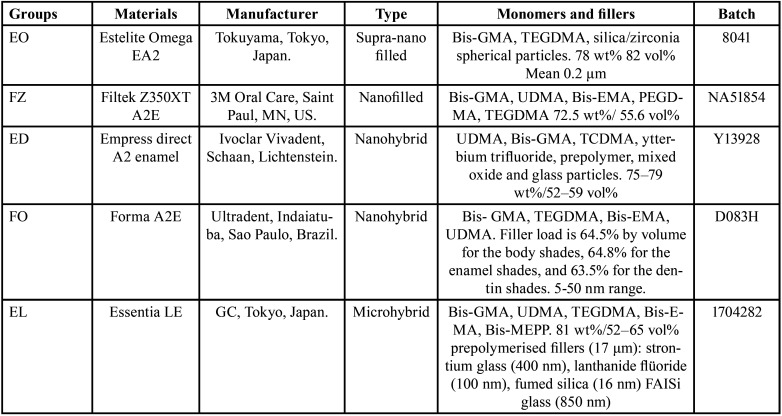
Groups and composition of materials tested [23-25].

Disc-shaped specimens (n=7) of each composite brand were prepared using a Teflon mold in a circular shape in increasing thickness (6 mm diameter X 3 mm thickness). The composite from each brand was filled in the mold, covered with glass plates in both directions, and filled in 2 different increments (light polymerized between increments). Light pressure was applied on both sides of the samples to eliminate the filling excess to obtain a smooth plane. All specimens were light polymerized for 40 seconds by a Spectrum Curing Light through the glass plates’ top and bottom. The samples were kept in distilled water at 37 ºC for 24 hours. After this period, the samples were removed from the mold and polished using a series of silicon carbide papers (600-, 1000-, and 1500-grits) to mimic clinical polishing systems (26).

-Translucency parameter

Two different backgrounds were used to calculate the translucency parameter (TP) and reproduce the discolored or stained tooth structures and the oral cavity’s darkness (27): a black and white background. The same background was used for all samples measured in the same room to ensure light standardization. A spectrophotometer (Vita Easyshade V; Vita Zahnfabrik) measured the samples’ colors and calibrated them according to the manufacturer’s instructions. Values were obtained according to the CIE L*a*b color system, the same used for the previously described test. The color difference (ΔE*ab) within the white and black backgrounds obtained the translucency parameter.

Statistical analysis was performed with a 1-way analysis of variance and a Bonferroni test to a 95% level of confidence.

-Fluorescence

From each group, one specimen at a time was placed in a box (with its inside walls painted black) under UV light (DW 54828, Damar). Afterward, a reflex digital camera, Nikon D90 with 105-mm f/2.8 Macro lens (Nikon Corporation, Tokyo, Japan), was used without the flash to capture an image of each specimen placed, in a predetermined position, inside the box. Ten photographs per group were taken. Subsequently, a digital photo editing program (Aperture 3.0, Apple, Inc.) was used to analyze each specimen’s central portion as shown on the digital photographic image, as previously described (28).

**Figure 1:**
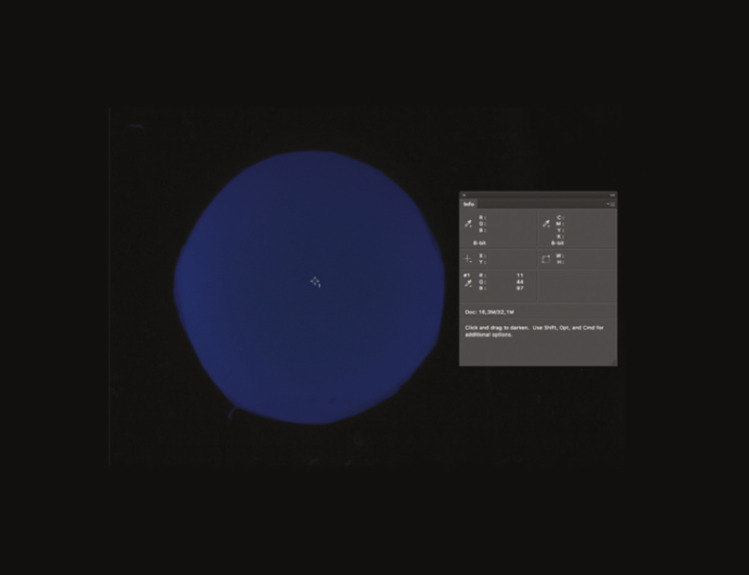
Digital photo editing program (Aperture 3.0, Apple, Inc.) analyzing the central portion shown on the digital photographic image.

The fluorescence analysis was conducted for each specimen as follows: each photograph was opened with the “Aperture software,” then the “Adjustments window” on the left side of the screen was selected, and a graph showing the values of red (R), green (G), and blue (B) was then displayed (Fig. 1). The Loupe tool was selected, setting it to “focus on loupe” and 100% expansion. Then this Loupe was positioned over the specimen pictures, and the cursor was placed at the center of the area to be evaluated. The B value (in the “Adjustments window”) was visualized in the screen’s left-side graphic. B shade’s brighter pattern in the evaluated image area corresponds to the specimen’s more significant fluorescence.

Statistical analysis was performed with the Kolmogorov-Smirnov test to check normality and mixed model analysis to a 95% confidence level to analyze group differences.

## Results

-Translucency parameter 

**Figure 2:**
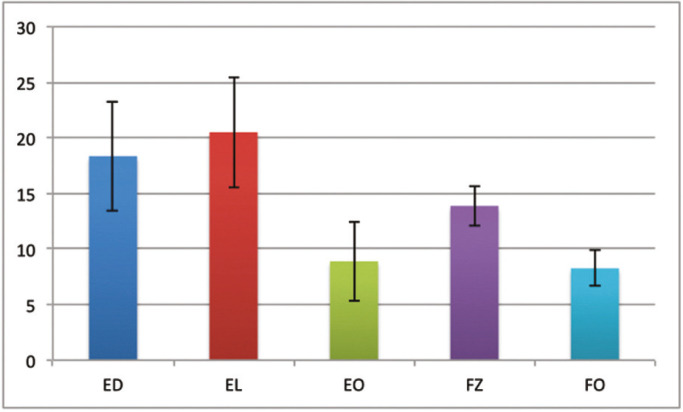
Graph showing translucency parameter values for each composite tested.

**Figure 3:**
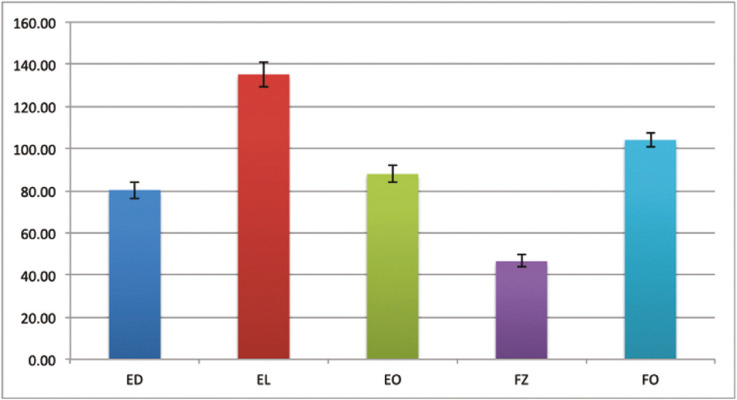
Graph showing mean fluorescence values for each composite tested.

The mean ∆E values ± standard error (SE) for the Translucency Parameter (TP) are described in Table 2 and graphed in (Figs. 2,3).

**Table 2:**
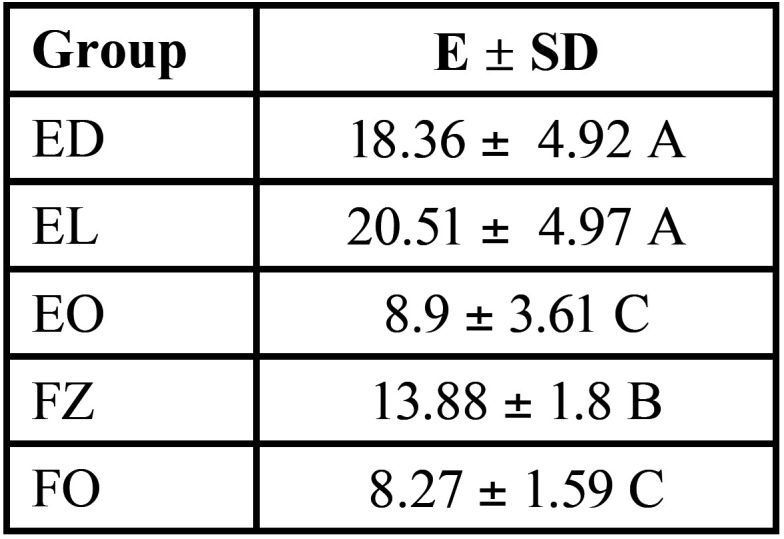
Mean ∆E values (Translucency parameter) with investigated composites’ standard deviations (SD). Means followed by different letters differ from each other in the same column (*p*<0.05).

The highest values were observed for ED and EL (*p*=0.00), followed by FZ (*p*=0.00). The lowest values were observed for EO and FO without statistical differences (*p*=0.69).

-Fluorescence

**Figure 4:**
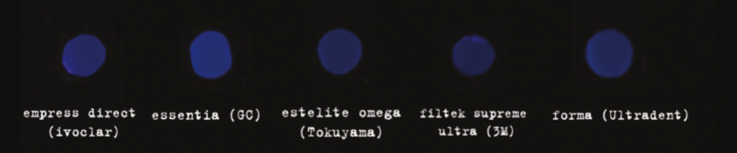
Composite groups under UV light in a dark box.

The mean values and standard deviation of Fluorescence Intensity (FI) for each group are given in Fig. 4 and Table 3.

**Table 3:**
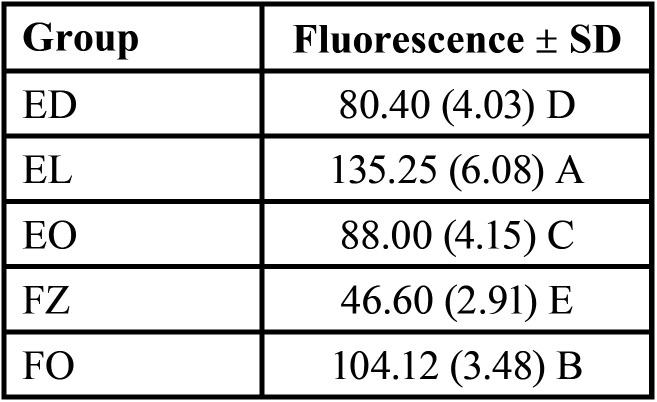
Mean fluorescence values with standard deviations (SD) of investigated composites. Means followed by different letters differ from each other in the same column (*p*<0.05).

The mean B Values ± SD of the resin composite was statistically different for all groups (*p*=0.000). The highest values were recorded for EL (135.25 ± 6.08), followed by FO (104.14 ± 3.45), EO (88.00 ± 4.15), ED (80.4 ± 4.03), and FZ (46.60 ± 2.91) (level of significance of 5%).

## Discussion

The null hypothesis was rejected once all groups showed differences in TP (translucency parameter) and the FI (fluorescence intensity).

The high translucency behavior of an enamel composite allows light to pass through the restorative material highlighting the chroma and opacity of the dentin layer used in the restoration (1). For an artificial enamel to reproduce the natural aspect of the tooth structure, adequate translucency is necessary.

TP or translucency parameter is defined as the color difference found for the material at a specified thickness. It is an alternative method compared to %T (Percentage of Translucency) measured by light transmittance (29). For TP, the color difference between the material in optical contact with ideal black and white backings is captured by a spectrophotometer (30). For samples with less than 2mm., the average TP was from 8 to 21 (31). Absolut TP values of materials tested obtained in our study are according to the literature (29).

EL group demonstrated a higher translucency parameter (20.51) than other groups but was not statistically different from ED (18.36). Both are statistically different from all other materials. This result reflects the highly translucent appearance of the enamel shades of both brands (El and ED). The reference of translucency should always be the natural human enamel that presents a TP of 18.7 in 1mm thickness (32), which is very close to the ED group of our study.

It has to be pointed out that the Essentia System (GC) doesn’t present a chromatic enamel like the other brands tested, so the EL (achromatic enamel) was used, which could partially explain the higher translucency obtained compared to other groups (1). The number of pigments of the “chromatic’ enamel A2 used for other brands could interfere with the translucency of these materials (33).

A translucency in enamel layers is fundamental to reproducing natural teeth’ optical properties and creating natural effects (1,5). Good opacity offered by dentin/opaque shades and translucency from enamel shades would allow a thriving esthetic outcome (34). From this perspective, a less translucent enamel composite like EO (TP 8.9) could produce a more opaque and artificial result than a more translucent material like EL or ED. The same result of less translucency (TP) of Estelite Sigma Quick, a similar composite to EO used in this test, compared to Empress Direct was obtained in other studies (29,35).

Translucency is related to the size and shape of the fillers, as well as the monomeric composition of the composite resin. An increase in the size and irregular shape of fillers could increase the scattering of the resin, decreasing the translucency (36), which could partially explain the lower values for FO that present more irregular fillers but couldn’t support the same low values for EO that has spherical and small filler particles. Other authors affirm that more minor fillers, like in the EO (20 nm.), could make complex the passage of the light inside the material (37), explaining the low TP obtained by EO. A higher amount of fillers with lower content of organic matrix should relate to a low TP, but this was also not supported by results once EL presented the higher translucency despite the high filler content (29). More research should be conducted on this matter to define a pattern.

The amount of BIS-GMA used in comparing UDMA and TEG-DMA could influence each composite system’s translucency level. BIS-GMA has a refractive index much closer to silica than other monomers (38). The refraction index of the resin matrix and the filler play a fundamental role in TP (39). Bis-GMA (RI = 1.54) has a refractive index similar to that of the silica filler (RI = 1.53) than that of TEGDMA (RI = 1.46); the difference in translucency may be attributable to this (39). Bis-EMA showed higher translucency compared to Bis-GMA and TED-GMA when compared to the composites formulated with the other base monomers(40). Still, even FO and FZ presenting Bis-EMA in composition, the translucency was low, maybe due to filler size (nanometric fillers and nano spherical particles).

The fluorescence of restorative material influences the value/brightness of the final restoration, becoming a vital behavior needed in an esthetic composite system. Nevertheless, some authors point out that natural teeth’ UV-induced fluorescence did not influence tooth color under standard daylight conditions (41).

Also, many patients, especially younger ones, complain about missing this brightness under UV light at parties and social events in restaurants, pubs, and nightclubs once these are the night lights in these environments.

Specific fillers in the resin formulation can control and increase the fluorescence of various restorative materials (15,16). The organic composition of resin composites also influences the intensity of the fluorescence (42). Manufacturers control technology and fabrication, keeping most of the information confidential. Resin matrix and filler dispersion could lead to less space for embedding the fluorescent pigments, which can be one cause of low values for a pure nanofiller composite like FS, with high particle dispersion inside the monomer (22).

Despite many techniques being used for the analysis of fluorescence behavior, like fluorescence spectrometer (17,18), photography attached to a UV illumination (19), direct spectrometry (20), monochromator-based multi-mode reader (21), among others, the use of digital photography measuring the intensity of composite resins is reliable and easy to be conducted, and had been used by other authors (19,43). Our test was conducted using UV light emission captured by a digital camera and data with specific analysis of values of red (R), green (G), and blue (B) displayed field (22).

Many wavelengths of excitation were tested (375, 395, and 410 nm), and they were similar for the three brands analyzed, with a maximum emission peak of emission at 450 nm (17). According to Meller & Klein (2012), the best emission peak was 398 nm (15). The Lamp used in this study emits a wavelength from 300 to 400 nm., with a peak I 368nm, the light usually used in nightclubs and bars (UV-A). The same light with a similar methodology was used in previous studies (44). Different composite thicknesses could interfere with the intensity of the fluorescence (18), but samples used in this study used 3mm—thicknesses to standardize results.

FI values are compatible with similar studies using the same methodology (19). Group EL composite showed the highest fluorescence value, significantly higher than other groups (135.25 ± 6.08). The reason for including enamel shades in this analysis is because the layer that offers final fluorescence in composite restorations is the enamel shade, different from the natural tooth, where the behavior comes from the dentin substrate (45). In this sense, the Light Enamel shade provided high final fluorescence.

The Fluorescence of all groups was different statistically under the UV light. Forma presented the second higher intensity of fluorescence, followed by EO and ED. The Absolut FI value obtained for ED was different from studies using the same methodology, probably due to light distance from the sample, the setting of the camera, and other variables (19).

In previous research, 3M Oraltech composites like Filtek Supreme and Z-250 had shown lower intensities of fluorescence peaks, following our results where group FZ (3M Oralcare) showed the lowest results of FI (2,17,21). A low fluorescence composite used as an enamel layer could produce a low luminosity restoration with an incorrect value/brightness and a more greyish effect (45). Other authors that had used composites compared to natural enamel concluded that despite having lower intensity peaks of fluorescence, 3M composites are closer to natural fluorescence than other brands (20). In our test, natural teeth weren’t used as a reference being a limitation of the study.

Clinically speaking, more fluorescence intensity (even higher to the natural teeth) could be positive, once aging decreases the fluorescence is about 70% after ten years. Fluorescence is also provided by organic matrix, so the hydrolysis of monomers could cause a decrease in fluorescence levels (42). In ceramic veneers, a highly fluorescent ceramic is used when an increase in luminosity is necessary without losing the translucency of the veneer when covering a dark background (46). From this perspective, a high-intensity fluorescence is attractive, independent of the intensity demonstrated by the natural tooth structure.

## Conclusions

With the limitations of this study, it can be concluded that:

The composition of fillers and organic matrix highly influenced the different behavior of fluorescence and translucency of resin composites.

EL and ED presented the highest translucency parameter, which was more similar to the natural human enamel.

The pure nano-filled composite (FZ) showed the lowest FI, while EL presented the highest.
